# Elevated monocyte HLA-DR in pediatric secondary hemophagocytic lymphohistiocytosis: a retrospective study

**DOI:** 10.3389/fimmu.2023.1286749

**Published:** 2023-11-24

**Authors:** Sylvain Raimbault, Guillaume Monneret, Morgane Gossez, Fabienne Venet, Alexandre Belot, Franck Zekre, Solene Remy, Etienne Javouhey

**Affiliations:** ^1^ Hospices Civils de Lyon, Hôpital Femme-Mère-Enfant, Service de Réanimation Pédiatrique, Bron, France; ^2^ Hospices Civils de Lyon, Hôpital Edouard Herriot, Laboratoire d’Immunologie, Lyon, France; ^3^ Hospices Civils de Lyon, Hôpital Femme-Mère-Enfant, Service de Néphrologie et Rhumatologie Pédiatrique, Centre de Référence RAISE (Rhumatismes Inflammatoires et Maladies Auto-Immunes Systémiques Rares de l’Enfant), ERN RITA (European Reference Network for Immunodeficiency, Autoinflammatory, Autoimmune and Paediatric Rheumatic Diseases), Bron, France

**Keywords:** hemophagocytic lymphohistiocytosis, monocyte HLA-DR, macrophage activation syndrome, lymphocyte activation, cytokine storm

## Abstract

**Introduction:**

Hemophagocytic lymphohistiocytosis (HLH) is a life-threatening condition, and its diagnosis may be challenging. In particular, some cases show close similarities to sepsis (fever, organ failure, and high ferritin), but their treatment, while urgent, differ: prompt broad-spectrum antibiotherapy for sepsis and immunosuppressive treatment for HLH. We questioned whether monocyte human leucocyte antigen (mHLA)–DR could be a diagnostic marker for secondary HLH (sHLH).

**Methods:**

We retrospectively reviewed data from patients with a sHLH diagnosis and mHLA-DR quantification. mHLA-DR data from healthy children and children with septic shock, whose HLA-DR expression is reduced, from a previously published study were also included for comparison.

**Results:**

Six patients with sHLH had mHLA-DR quantification. The median level of monocyte mHLA-DR expression in patients with sHLH [79,409 antibodies bound per cell (AB/C), interquartile range (IQR) (75,734–86,453)] was significantly higher than that in healthy children and those with septic shock (29,668 AB/C, IQR (24,335–39,199), and 7,493 AB/C, IQR (3,758–14,659), respectively). Each patient with sHLH had a mHLA-DR higher than our laboratory normal values. Four patients had a second mHLA-DR sampling 2 to 4 days after the initial analysis and treatment initiation with high-dose corticosteroids; for all patients, mHLA-DR decreased to within or close to the normal range. One patient with systemic juvenile idiopathic arthritis had repeated mHLA-DR measurements over a 200-day period during which she underwent four HLH episodes. mHLA-DR increased during relapses and normalized after treatment incrementation.

**Conclusion:**

In this small series, mHLA-DR was systematically elevated in patients with sHLH. Elevated mHLA-DR could contribute to sHLH diagnosis and help earlier distinction with septic shock.

## Introduction

Hemophagocytic lymphohistiocytosis (HLH) is a life-threatening condition associated with unregulated and deleterious T-lymphocyte and macrophage activation. Various genetic and environmental factors may cause HLH through a defective immune regulation (1); these are commonly separated between primary/genetic HLH and secondary forms [sHLH; associated with rheumatologic diseases termed macrophage activation syndrome (MAS), malignancies, viral infections, and primary immunodeficiencies but also drug-induced (2, 3)]. HLH diagnosis may be challenging because of heterogeneity in cause and clinical presentation (4, 5), and this has led to the development and validation of scores adapted to specific populations (HLH-2004, 2016 EULAR/ACR, HScore) that are based on common parameters but with different thresholds (1, 6, 7).

Recent reports have explored markers of T-cell activation and found that HLH was characterized by strongly elevated activated CD8^+^ T cells. Specifically, CD38^high^ HLA-DR^+^ CD8^+^ T cells above 7% (among CD8^+^ T cells) could help differentiate HLH from sepsis (8). Similarly, sHLH is associated with a proportion of CD4^dim^ CD8^+^ T cells (that are highly activated and cytolytic CD8^+^ mature T cells) above 1.45% that reliably discriminate sHLH from other causes of systemic inflammation such as flare of systemic juvenile idiopathic arthritis (sJIA) (9). HLA-DR expression on T cells could also help to identify primary from sHLH with a higher expression in primary forms (10). However, no marker of monocyte activation has been described in HLH, although medullar hemophagocytosis, elevated Interferon-γ (IFN-γ), and serum biomarker profile (11–13) suggest a central role of macrophage stimulation in HLH pathogenesis. The two main actions of IFN-γ are, on the one hand, to enhance CD8^+^ T-cell cytotoxicity and T-helper 1 (Th1) response; and, on the other, to increase monocyte/macrophage activity and their expression of MHC surface molecules together with an enhancement of their phagocytic capacity. HLA-DR expression on monocytes reflects IFN-γ plasma levels (14). In 2018, we published the case of a young adolescent female patient suspected of septic shock (15), who was included in a prospective cohort of sepsis immunomonitoring: PedIRIS (16). The unexpectedly high level of monocyte human leucocyte antigen (mHLA)–DR (137,021 antibodies bound per cell, AB/C) compared to the usual low value of patients with septic shock (below 10,000 AB/C) helped in the diagnosis of sHLH caused by drug-induced hypersensitivity syndrome (15).

This raises the question of whether mHLA-DR could contribute to sHLH diagnosis, and following this case mHLA-DR was added to the usual lymphocyte phenotyping conducted upon sHLH suspicion in patients admitted to the pediatric departments of our institution. Herein, we retrospectively analyzed the clinical and laboratory data of these patients to describe mHLA-DR levels among children with sHLH diagnosis.

## Materials and methods

### Patients

We included all patients admitted to the pediatric intensive care unit or the pediatric nephrology and rheumatology department (Hospices Civils de Lyon, Lyon, France) who had a diagnosis of sHLH according to the 2016 EULAR/ACR classification criteria (6) and at least one mHLA-DR quantification between March 2018 and June 2022. The patient previously reported (15) was also included in the present study.

### Clinical and laboratory data

We recorded for each patient their medical history, the date of sHLH diagnosis, their clinical data (fever, presence of splenomegaly, previous treatments, as well as those for the current episode), and the following laboratory data: ferritin, triglycerides, fibrinogen, aspartate amino-transferase (ASAT), hemoglobin, platelet count, white blood cell count, and evidence of bone marrow hemophagocytosis.

The values for all presented variables correspond to those on the day of maximum ferritin for each patient; in cases where these data were not available, the values obtained within a 24-h period (3 days for bone marrow aspiration) were recorded (and in case of multiple tests, the closest was considered). Expression of mHLA-DR was measured using flow cytometry following a previously published protocol and expressed as AB/C (17). In addition mHLA-DR data from healthy children and children with septic shock from a study previously published (16) were also included for comparison. Healthy children had a median age of 2.8 years [interquartile range (IQR) (1.5–5.9)], and 8 (27%) were female patients; children with septic shock had a median age of 2.4 years [IQR (0.4–4.4)], and 24 (52%) were female patients.

### mHLA-DR flow cytometry measurement

The number of HLA-DR molecules per monocyte was determined using the BD quantibrite standardized method. Briefly, 25 μL of whole blood were stained with 10 μL of QuantiBrite HLA-DR/Monocyte mixture [QuantiBrite anti–HLA-DR Phycoerythrin (PE) (clone L243)/anti-monocytes (CD14) PerCP-Cy5.5 (clone MϕP9), Becton Dickinson San Jose, CA, USA] and 5µl of antiCD19 Pacific blue (PB) and kept at room temperature for 30 min in a dark chamber. Samples were then lysed with Fluorescence-Activated Cell Sorting (FACS) Lysing solution (Becton Dickinson) for 15 min. After a washing step, cells were analyzed on Navios Cytometer (Beckman Coulter). After leukocyte isolation on a Forward Scatter/Side Scatter biparametric chart to exclude possible red cells, monocytes and B lymphocytes were gated out, respectively, on the basis of CD14 and CD19 expression. HLA-DR expression on monocyte was expressed on a monoparametric chart, and its mean fluorescence intensity (MFI) was measured. B lymphocytes were removed from the gated monocytes because of their high HLA-DR expression to avoid faulty increase of mHLA-DR MFI.

To enable standardized results, mHLA-DR MFI was converted into the number of anti–HLA-DR antibody per cell (AB/C), using calibration beads (QuantibriteH, Becton Dickinson). Results were then expressed as the number of anti–HLA-DR antibodies per cell (AB/C). We ran monthly assays with calibration beads for conversion from MFI to AB/C (18, 19).

### Statistical analysis

Patient characteristics and laboratory values were described by frequency, median, and IQR. Comparisons between sHLH, healthy children, and those with septic shock were conducted using Mann–Whitney two-sided unpaired test. Data analysis was performed using R software for Windows, version 4.2.2 (The R Foundation for Statistical Computing, Vienna, Austria).

## Results

A total of six patients were included. The median age was 14.1 years [IQR (7.5–16.1)], and the etiology of sHLH was Epstein-Barr virus–induced for one patient, rheumatological in the course of sJIA for two patients, drug-induced for one patient, and unknown for the remaining two patients (**Table 1**). Both the latter had a history of persistent unexplained fever, for which possible infections had been ruled out (2016 EULAR/ACR criteria). Symptomatology resolved in these patients, and laboratory parameters normalized, but the cause of sHLH was not documented; no recurrence was observed during follow-up.

**Table 1:** Clinical and laboratory data of HLH patients.

Patient	1	2	3	4	5	6	Total^a^
Age, years	16.2	17.5	5.9	15.9	1.6	12.4	14.1 (7.5–16.1)
HLH etiology	Drug-induced	sJIA	sJIA	Uk	Infectious	Uk	
mHLA-DR, AB/C	137,021	88,500	46,124	74,809	78,509	80,308	79,409 (75,734–86,453)
MODS	Yes	No	No	No	No	No	17%
Splenomegaly	Yes	No	Yes	Yes	Yes	No	67%
Fever	Yes	Yes	Yes	Yes	Yes	Yes	100%
Ferritin, µg/L (NV: 5–100)	3,918	8,603	26,888	9,024	19,262	1,905	8,814 (5,089–16,703)
Triglycerides, mmol/L (NV: 0.4–1.7)	1.82	1.82	1.40	1.18	4.82	4.27	1.82 (1.51–3.66)
ASAT, U/L (NV: <55)	712	221	128	478	876	265	372 (232–654)
Fibrinogen, g/L (NV: 1.64–4.97)	0.71	NA	3.50	0.68	1.57	2.41	1.57 (0.71–2.41)
Hemoglobin, g/L	109	128	86	106	107	100	107 (97–114)
Platelets, G/L	120	242	552	74	114	265	181 (104–337]
Granulocytes, G/L	11.3	2.3	4.3	0.5	2.5	0.8	2.4 (0.7–6.1)
Hemophago-cytosis	No	NA	Yes	Yes	Yes	NA	75%

Indicated normal values (NV) correspond to our laboratory’s. They are not indicated for blood cell counts as normal values vary with age.

AB/C, antibody bound per cell; ASAT, aspartate amino-transferase; HLH, hemophagocytic lymphohistiocytosis; IQR, interquartile range; mHLA-DR, monocyte human leucocyte antigen–DR; MODS, multiorgan deficiency syndrome; NA, not available; NV, normal values; sJIA, systemic juvenile idiopathic arthritis; Uk, unknown.

a Expressed in median (IQR) or %.

All patients presented with fever. Splenomegaly was found in four (67%) patients. One (Patient 1) had multiorgan deficiency syndrome (MODS) with hemodynamic instability requiring vasopressor therapy and respiratory distress with mechanical ventilation. Ferritin levels were elevated, and the median value was 8,814 µg/L [IQR (5,089–16,703)]; ASAT levels were also elevated, and the median value was 372 U/L [IQR (232–654)]. Triglyceride and fibrinogen levels were not abnormal in all patients, and the median triglycerides value was 1.82 mmol/L [IQR (1.51–3.66)] and that of fibrinogen was 1.57 g/L [IQR (0.71–2.41)]. Cytopenia was found in all patients, with monocytopenia for two patients, bicytopenia for two patients, and pancytopenia for the remaining two patients. Bone marrow aspiration found evidence of hemophagocytosis in three of the four patients for which a sample had been taken (**Table 1**).

Only the two patients with sJIA-associated HLH had an ongoing treatment prior to mHLA-DR measurement. Patient 2 had a systemic corticosteroid therapy for 3 months that was progressively tapered to 0.3 mg/kg at the time of sampling, and anti-Interleukin-1 (IL1) receptor agonist (anakinra), started 2 weeks earlier. Patient 3 had a high-dose intravenous immunoglobin treatment (twice 1 g/kg) in the preceding 2 weeks.

**Figure 1:**
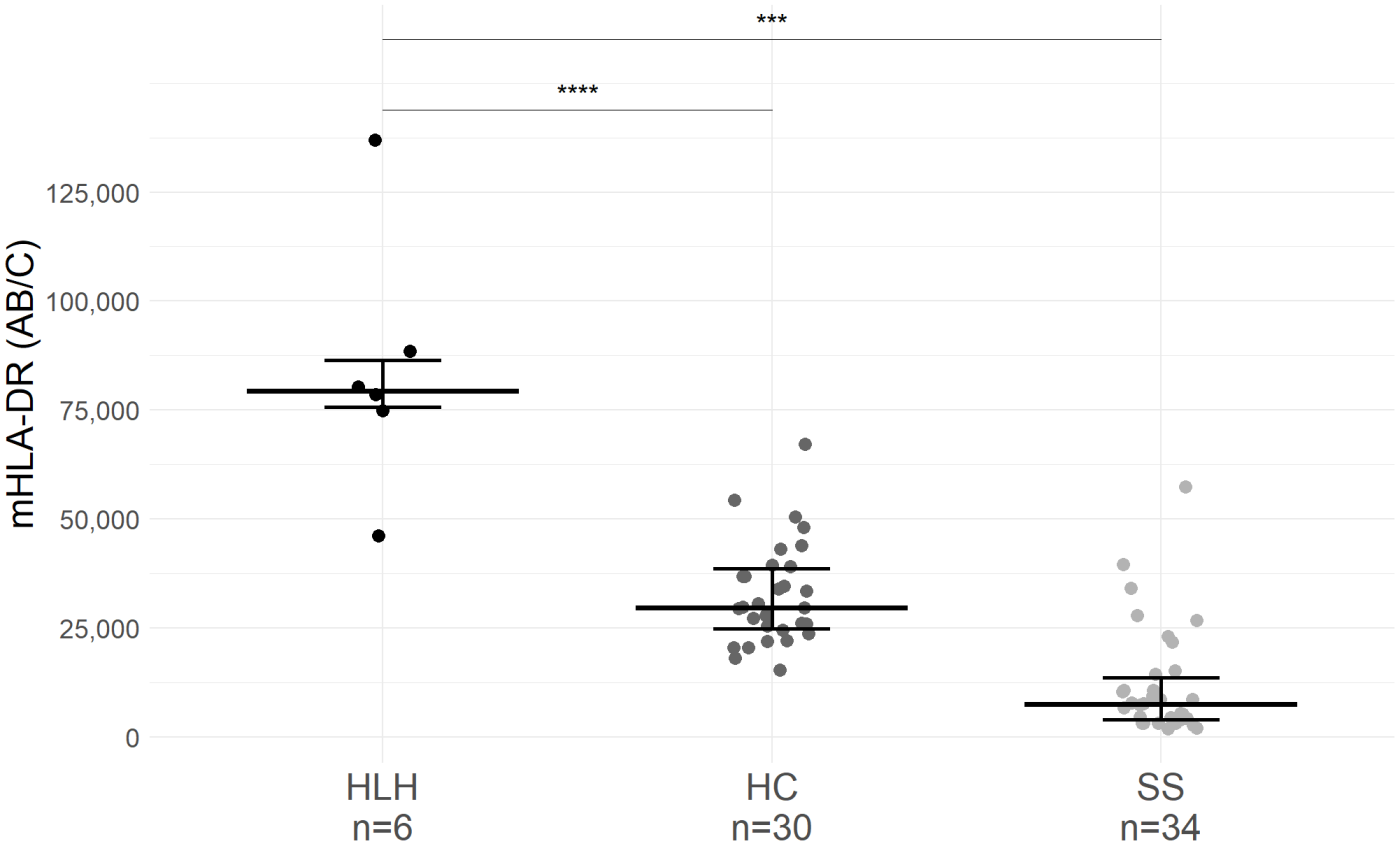
mHLA-DR expression in patients with hemophagocytic lymphohistiocytosis (HLH), healthy children (HC), and septic shock (SS). Only the first mHLA-DR value was retained for each patient. Values of mHLA-DR from HC and patients with SS originated from a previous study conducted in our institution [NCT02848144 (14)]. mHLA-DR expression corresponds to the number of antibodies bound per cell (AB/C). Wide lines correspond to the median value and narrow lines to first and third quartiles. Median age of HC was 2.8 years old [IQR (1.5–5.9)], and median age of SS children was 2.4 years old [IQR (0.4–4.4)]. Dots correspond to individual values. *** p<0.01, **** p<0.001.

The median level of monocyte mHLA-DR expression in patients with sHLH [79,409 AB/C, IQR (75,734–86,453)] was significantly higher than in healthy children [29,668 AB/C, IQR (24,335–39,199), p < 0.01] and in children with septic shock [7,493 AB/C, IQR (3,758–14,659), p < 0.01]. Each patient with sHLH had a mHLA-DR higher than our laboratory normal values (15,000–43,000 AB/C). Only one patient with sHLH (Patient 3) had a mHLA-DR value (46,124 AB/C) overlapping with that of the healthy children (maximum of 67,255 AB/C; **Figure 1**).

**Figure 2:**
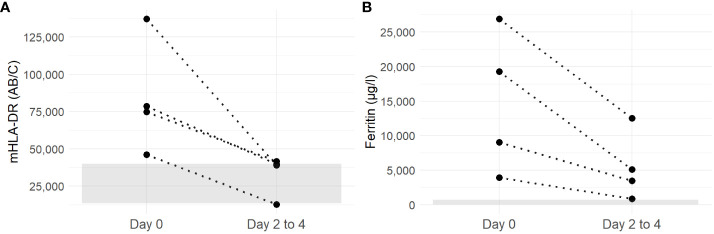
Evolution of mHLA-DR **(A)** and ferritin **(B)** of patients with sHLH between day 0 (before corticosteroid initiation) and days 2 to 4 (after corticosteroid treatment). Discontinuous lines link values from each individual patient. The gray ribbon corresponds to the laboratory normal values for mHLA-DR and ferritine (mHLA-DR: 15,000–43,000 AB/C).

Four patients (Patients 1, 3, 4, and 5) had a second mHLA-DR measurement 2 to 4 days after the initial analysis and treatment initiation with high-dose corticosteroids; for all four patients, the values decreased to within or close to the normal range (**Figure 2A**). Ferritin paralleled this favorable course, although values remained above the normal range (**Figure 2B**). Normalization of all HLH laboratory criteria was achieved for each patient within 30 days.

**Figure 3:**
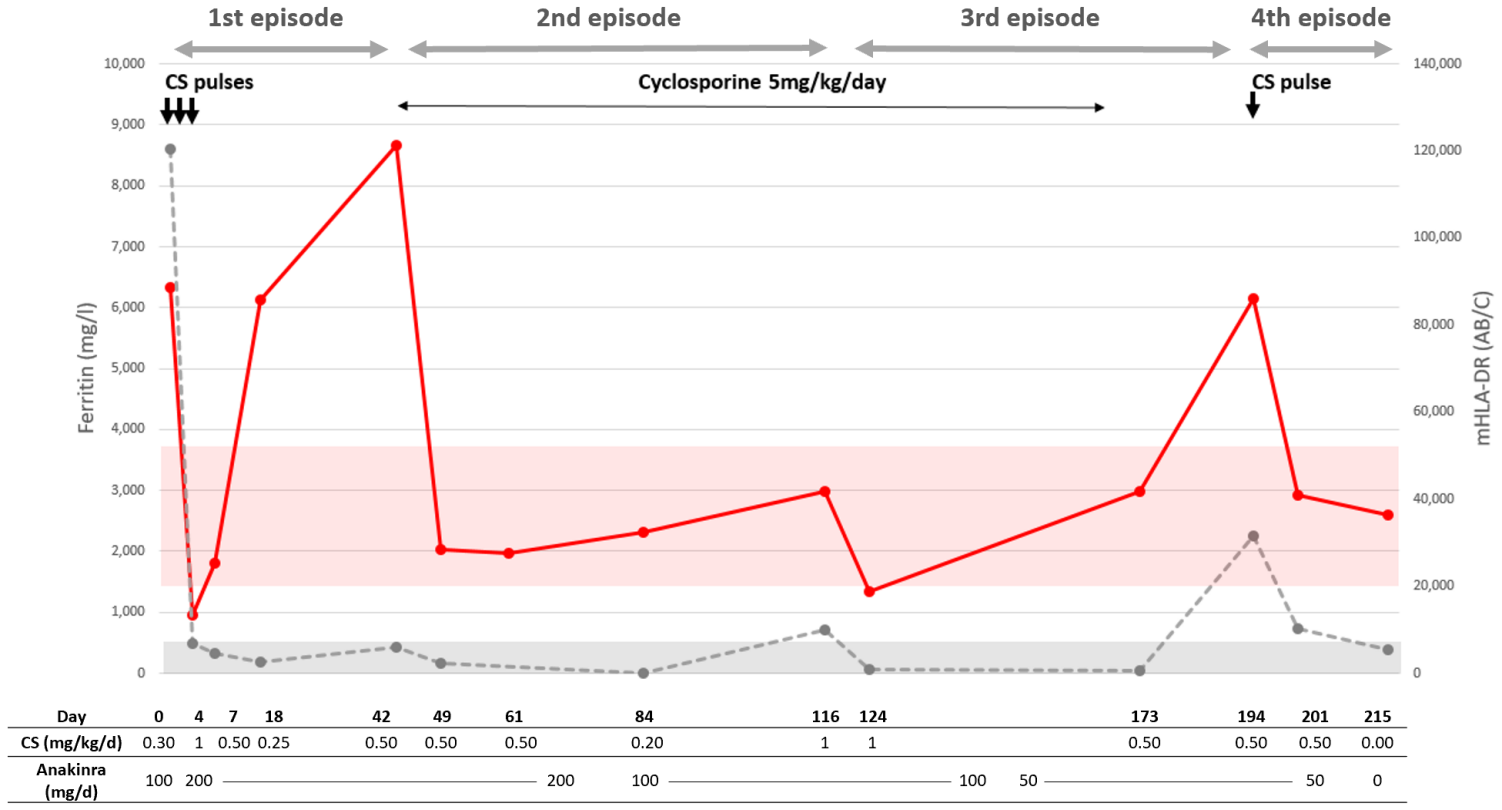
Course of Patient 1. Red dots and line correspond to mHLA-DR; the red ribbon corresponds to normal values. The gray dots and discontinuous line correspond to ferritin; the gray ribbon corresponds to normal values with an upper threshold of 500 µg/L. Each corticosteroid (CS) pulse arrow corresponds to methylprednisolone at 10mg/kg. The start of a new episode is defined by the relapse of sHLH symptoms and laboratory criteria.

One patient (Patient 2) had repeated mHLA-DR measurements over a period of over 200 days, during which she underwent four episodes of rheumatologic-associated MAS (**Figure 3**). She was a 17-year-old female patient with a history of sJIA, diagnosed 1 month before the first occurrence of MAS. She was treated with anakinra (IL1 receptor antagonist, 100 mg/day) and oral corticosteroid therapy that was being tapered (0.3 mg/kg/day at the time of MAS). The first MAS episode was suspected clinically because of persistent fever and fatigue. Her ferritin level was elevated (8,603 µg/L), and she had a mild hypertriglyceridemia (1.82 mmol/L) and hepatic cytolysis (ASAT: 221 U/L); mHLA-DR was markedly elevated (88,500 AB/C). After treatment with three consecutive corticosteroid pulses at 10 mg/kg, increase of anakinra to 200 mg/day and of oral corticosteroids, a strong decrease in both ferritin and mHLA-DR was observed. Fever and fatigue resolved. A month and a half later while corticosteroids were being tapered, despite a favorable clinical course, ferritin started to rise slowly and mHLA-DR had risen dramatically. To avoid excessive cumulative corticosteroid treatment, cyclosporine was started at 5 mg/kg/day with a favorable effect on ferritin and mHLA-DR. Less than 3 months after cyclosporine initiation, after a decrease of oral corticosteroids to 0.2 mg/kg/day and of anakinra to 100 mg/day, fever resumed along with fatigue and joint pain. Ferritin had risen to 714 µg/l, as did mHLA-DR but below the upper normal range (41,731AB/C). An increase in oral corticosteroid treatment to 1 mg/kg/day was sufficient to improve clinical and laboratory signs of disease activity. Similarly, 2 months later, and in the absence of cyclosporine and while anakinra and corticosteroid treatment were being tapered, she experienced a fourth MAS episode. She suffered from abnormal fatigue and fever for 7 days. Analyses found signs of MAS: ferritin at 2256 µg/L and ASAT at 101U/L; mHLA-DR was elevated (86,005 AB/C). After treatment with one corticosteroid pulse and a moderate increase in oral corticosteroids, both clinical and laboratory signs of MAS normalized. She did not experience another episode of MAS, but corticodependance remained elevated thereafter.

## Discussion

In the present study, mHLA-DR levels were systematically abnormally high in patients with sHLH, and, in contrast to other HLH biomarkers such as ferritin, mHLA-DR quickly normalized under corticosteroid treatment and conversely rose in case of sHLH relapse. In this context, mHLA-DR might be a useful tool for their differential diagnosis, reflecting the known physiopathology of HLH driven by IFN-γ production, leading to an enhanced and dysregulated activation of CD8^+^ cytotoxic T cells and monocytes. In addition, among patients with MODS, mHLA-DR could be able to better distinguish sHLH from those with another etiology than the majority of HLH markers. A low mHLA-DR level has been well established as a marker of monocyte anergy in adult patients who experience septic shock (20), severe trauma, and burns or who undergo cardiac surgery with extra-corporeal circulation (21), and similar results are reported in pediatric populations (16, 22–24); herein, the patient with MODS, initially considered as a septic shock, had extremely elevated mHLA-DR (three-fold), and it was later understood that the etiology was a drug reaction with eosinophilia and systemic symptoms associated to HLH, and treatment with corticosteroids was sufficient for a resolution of clinical symptoms and laboratory parameters (15).

sHLH is a complex and multifaceted syndrome, and its diagnosis remains a challenge to this day, overlapping with other cytokine storm such as sepsis (25–27). The validated scores adapted to specific populations are based on the same laboratory parameters (high ferritin, high triglycerides, low fibrinogen, presence of cytopenia, and elevated ASAT) but use different thresholds. For instance, analyses of the performance of HLH-2004 in the setting of adult ICU found that changing ferritin and fever cutoffs was necessary to take into account the other causes of lesser ferritin elevation and fever among ICU patients, such as those with sepsis (28), and which led to the development of the Hscore (7). It is of note that, despite being eponymous of sHLH, hemophagocytosis does not have either a good positive or a good negative predictive value as it is associated with many conditions (29), especially within a critical care setting, as highlighted by a postmortem evaluation of critically ill patients that found less than two-thirds of patients had hemophagocytosis (30). This issue is all the more important considering that, while sharing symptomatic treatment of organ dysfunction, etiologic treatment differ; a prompt and broad-spectrum antibiotherapy is required for sepsis, but an effective immunosuppression with high-dose corticosteroids, possibly in association with etoposide or cyclosporine, is required for sHLH (1, 31–33). The data presented herein therefore suggest that mHLA-DR could contribute to sHLH diagnosis. However, the study presents several limitations. Because of the retrospective nature of the study design, mHLA-DR and other laboratory values were not systematically determined on the same day. In addition, sHLH diagnosis and etiology required a *post-hoc* analysis to be confirmed which exposes to a risk of misclassification. In addition, because the pediatric hematology department is in another institution in Lyon, the cohort does not include hemopathy-associated HLH that may present with differences regarding mHLA-DR levels. A more general note is that the small sample included herein may not represent accurately HLH patients as a whole and precludes the investigation of a mHLA-DR cutoff for sHLH diagnosis. A prospective study is therefore warranted to further investigate mHLA-DR as a marker for sHLH, with a larger cohort of patients, and a balanced distribution between HLH etiologies, including primary HLH and hemopathy-associated HLH.

In conclusion, elevated mHLA-DR could contribute to sHLH diagnosis, but this requires further investigation in a larger study.

## Data availability statement

The raw data supporting the conclusions of this article will be made available by the authors, without undue reservation.

## Ethics statement

The studies involving humans were approved by Scientific and Ethical Committee of Hospices Civils de Lyon. The studies were conducted in accordance with the local legislation and institutional requirements. The ethics committee/institutional review board waived the requirement of written informed consent for participation from the participants or the participants’ legal guardians/next of kin because the data used originated from medical files and was made anonymous prior to analysis. Patients and legal guardians are informed during their stay that medical data may be used for retrospective analysis.

## Author contributions

SyR: Conceptualization, Data curation, Formal analysis, Investigation, Methodology, Writing – original draft, Writing – review & editing. GM: Methodology, Supervision, Validation, Writing – review & editing. MG: Data curation, Methodology, Writing – review & editing. FV: Data curation, Methodology, Validation, Writing – review & editing. AB: Methodology, Supervision, Writing – review & editing. FZ: Data curation, Validation, Writing – review & editing. SoR: Data curation, Validation, Writing – review & editing. EJ: Conceptualization, Methodology, Validation, Writing – review & editing.
